# A watch-and-wait approach for metachronous multiple colon cancer following neoadjuvant immunotherapy: a case report

**DOI:** 10.3389/fimmu.2024.1391038

**Published:** 2024-10-04

**Authors:** Wang Huang, Shouru Zhang

**Affiliations:** Department of Gastrointestinal Surgery, Chongqing University Cancer Hospital, Chongqing, ;China

**Keywords:** advanced colon cancer, neoadjuvant immunotherapy, microsatellite instability high, clinical complete response, wait-and-watch, lynch syndrome

## Abstract

The application of immunotherapy for treating colorectal cancer (CRC) is currently a research hotspot, and neoadjuvant immunotherapy has shown initial success in treating CRC. The watch-and-wait (W&W) approach is often used after achieving a clinical complete response (cCR) following preoperative treatment of low rectal cancer. However, thus far, the W&W approach has not been reported for patients with colon cancer. Here, we report the case of a 64-year-old patient with heterogeneous multigenic CRC who achieved cCR after five sessions of neoadjuvant immunotherapy before surgery. A W&W approach was used to spare the patient from surgery. A 64-year-old male presented with intermittent abdominal pain. A colonoscopy examination detected an irregular cauliflower-like mass near the hepatic flexure of the ascending colon. The biopsy results indicated adenocarcinoma of the ascending colon. The patient was administered pembrolizumab (200 mg, ivgtt, q3w). After one cycle of treatment, the intestinal obstruction symptoms disappeared, and the treatment was continued for additional three sessions. After complete clinical remission of the tumor was confirmed, the W&W approach was adopted. Follow-up CT scans and colonoscopy examinations confirmed no local tumor regeneration or metastasis. Neoadjuvant immunotherapy is effective for patients with DNA mismatch repair gene deficiency and/or microsatellite instability high with a high rate of cCR or pathologic complete response. The W&W approach may also be suitable for patients with colon cancer. The safety and feasibility of watch and wait in patients with colon cancer need to be verified by more clinical data.

## 1 Introduction

CRC is one of the most common malignant tumors, and its incidence and mortality rank third and second, respectively, among all cancer (1, 2). CRC is mainly treated by surgery, radiotherapy, and systemic therapy, including chemotherapy, targeted therapy, and immunotherapy. Among these therapies, immunotherapy is one of the latest and most promising treatment methods. Recent research and breakthroughs in cancer therapy have considerably changed the treatment strategy for gastrointestinal tumors.

Previous studies have shown that 10–20% of CRCs have microsatellite instability (MSI) (3). CRC patients with DNA mismatch repair (dMMR) gene deficiency and/or microsatellite instability high (MSI-H) are the main beneficiaries of immunotherapy; moreover, neoadjuvant immunotherapy has shown initial success in the preoperative treatment of these patients. Thus far, the remission rate of neoadjuvant immunotherapy in these patients is almost 100%, and the pathological complete response (pCR) rate is >60%. Neoadjuvant immunotherapy can achieve sustained clinical complete remission that could enable organ preservation and exclusion from surgery as well as avoid the adverse effects of radiotherapy or surgery on defecation, urination, and sexual functions and fertility.

Currently, for patients with low rectal cancer who achieve clinical complete response (cCR) following neoadjuvant immunotherapy, concurrent chemoradiotherapy, total neoadjuvant chemoradiotherapy, or chemoradiotherapy combined with immunotherapy, the watch-and-wait (W&W) approach is often used to achieve anal preservation and exclusion from surgery. However, for patients with colon cancer, it is generally believed that radical surgery is necessary even after cCR is achieved.

Here, we report the case of a metachronous CRC patient with MSI-H and lynch syndrome (LS) who was successfully treated with neoadjuvant immunotherapy. The use of the W&W approach improved the patient’s quality of life and avoided the risk of surgery. Thus far, local regeneration and metastasis have not been observed in the patient. This case report presents a new strategy for the neoadjuvant treatment of advanced colon cancer.

## 2 Case report

A 64-year-old male presented to our hospital on March 27, 2023, with the complaint of intermittent abdominal pain for the last 8 months. Eight months earlier, the patient had experienced intermittent episodes of abdominal distention and pain. The condition mainly manifested as abdominal distension and pain after eating solid foods. The patient felt dull pain and experienced pain relief after defecation. Following treatment with Weisen and infusion, the pain was alleviated. The patient had no nausea, vomiting, and other discomfort. However, the patient experienced more recurrent episodes of abdominal distension and pain, but did not undergo treatment. Twenty-one days ago, the patient was admitted to another hospital; clinical examination indicated that the ascending colon wall was thickened; tumor recurrence was considered, and further examination was proposed. The patient was sent to a local hospital in Guizhou for treatment. A colonoscopy examination detected an irregular cauliflower-like mass near the hepatic flexure of the ascending colon; however, further examination could not be performed as the passage of the colonoscope was blocked due to a narrow lumen. The biopsy results indicated adenocarcinoma of the ascending colon.

On May 07, 2015, the patient underwent laparoscopic radical resection of rectal adenocarcinoma in another medical center. The postoperative diagnosis was a pT4N0M0 (stage II) tumor. No specific postoperative disease was detected, and chemotherapy was administered 4 times after surgery. The patient then underwent ileocecal resection in the same medical center on December 07, 2016. Postoperative findings were as follows: (ileocecal) tubulovillous adenoma with high-grade intraepithelial neoplasia, focal intramucosal carcinoma, no cancerous mass in the ileum, no resection margin of the colon and appendix, and no cancerous mass in the mesenteric lymph node (0/8). The patient underwent four sessions of chemotherapy after surgery; details regarding two chemotherapy regimens and doses were missing. Subsequently, gastroenteroscopy and CT scan findings were reviewed each year, and intestinal adenoma resection was performed several times. Tumor recurrence and metastasis were not detected, and no review was conducted in the past 2 years. The patient had no history of hypertension and diabetes. Moreover, although the patient had a smoking history, he was a nondrinker.

Physical examination on admission showed an Eastern Cooperative Oncology Group (ECOG) score of 1. Abdominal examination revealed old surgical scars, slightly active intestinal sounds (approximately 6 times per minute), and sound of passage of air and water. The remaining features were normal.

Auxiliary examination: On March 6, 2023, a CT scan conducted in the medical center of another hospital (The patient had undergone two intestinal surgeries at this hospital) showed that the ascending colon wall was thickened; tumor recurrence was considered, and further examination was recommended. On March 21, 2023, a colonoscopy examination conducted in a local hospital in Guizhou revealed an irregular cauliflower-like mass in the ascending colon near the hepatic flexure. The lumen was narrow and blocked the passage of the colonoscope mirror. The tumor material was friable and easily bled upon contact. The biopsy result suggested adenocarcinoma of the ascending colon.

**Figure 1:**
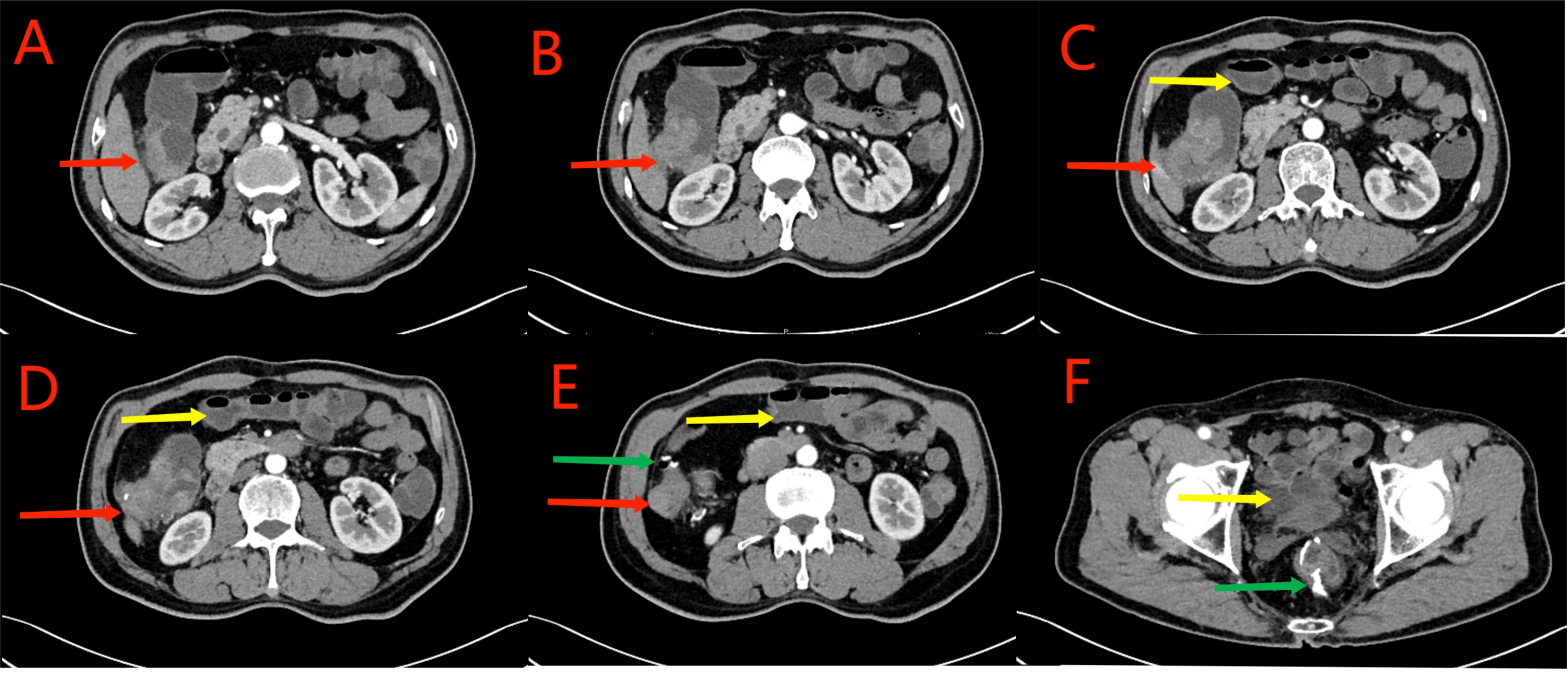
Red arrows in **(A–D)** show the ascending colon tumor, multiple enlarged lymph nodes in the surrounding adipose space, and suspected liver invasion. Yellow arrows in **(C–F)** indicate the dilated small intestine filled with gas and fluid. Partial small intestinal obstruction was considered. Green arrows in **(E, F)** show the previous post-cecal anastomosis and post-rectal anastomosis, respectively.

**Figure 2:**
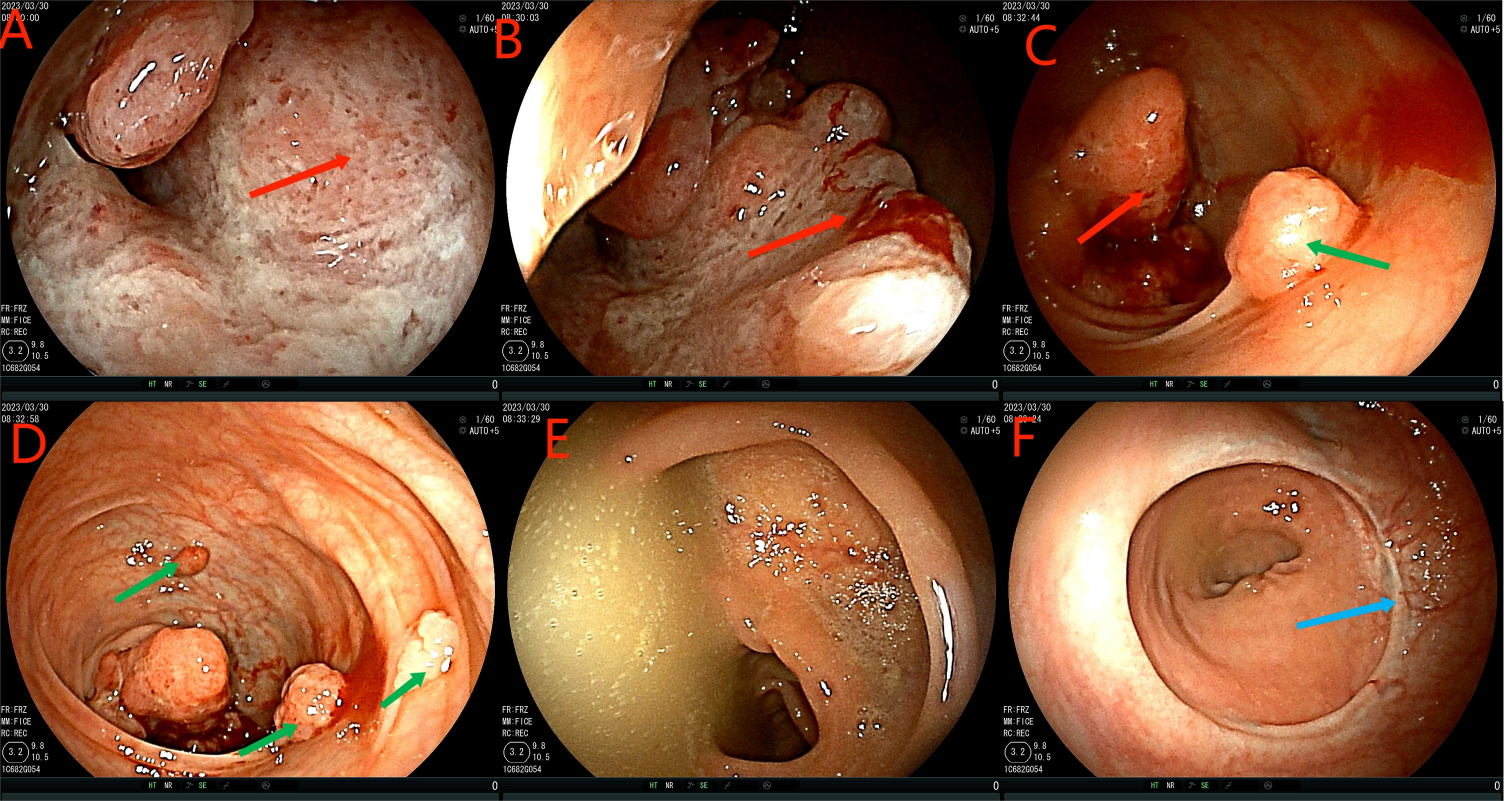
Colonoscopy examination time is shown in the upper left corner of each image. The red arrow in the **(A–C)** figure is the ascending colon tumor, which cannot pass through the colonoscope. Green arrows in figures **(C, D)** indicate multiple adenomas near intestinal tumors. The **(E)** picture shows fecal water overflowing at the proximal end of the tumor during the process of endoscopy, indicating incomplete intestinal obstruction. **(F)** is a rectal anastomosis.

**Figure 3:**
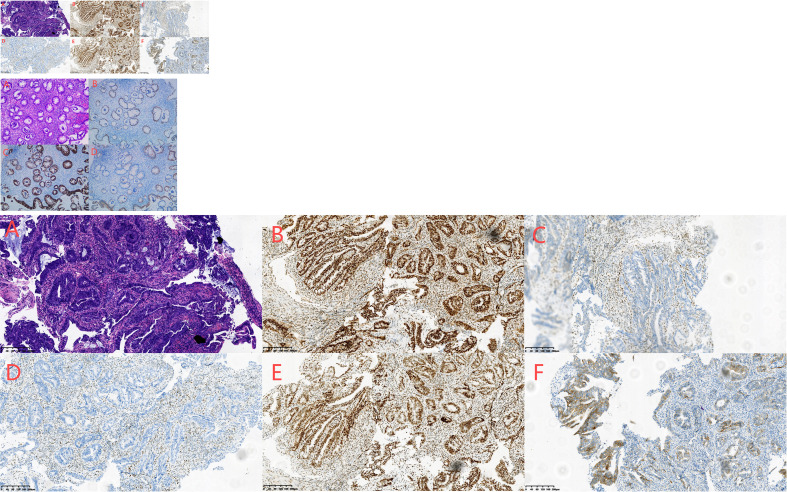
**(A–F)** Biopsy of adenocarcinoma of the ascending colon conducted on April 4, 2023. HE staining showed irregular, twisted, co-mural or back-to-back glands, partially sieve shaped, moderate to severe cell atypia, focal with more mucus secretion **(A)**. Immunohistochemical staining showed the following results:MLH1 (+) **(B)**, MSH2 (-) **(C)**, MSH6 (-) **(D)**, PMS2 (+) **(E)**, her-2 (2 +) **(F)**, P53 (+ 60%) and Ki-67 (+ 80%).

Relevant laboratory tests were completed after admission, as shown in **Table 1**. Following admission, a CT scan (**Figure 1**) and a colonoscopy examination (**Figure 2**) were conducted, and biopsy (**Figure 3A**) showed adenocarcinoma of the ascending colon. Immunohistochemical staining showed the following results:MLH1 (+) (**Figure 3B**), MSH2 (-) (**Figure 3C**), MSH6 (-) (**Figure 3D**), PMS2 (+) (**Figure 3E**), her-2 (2 +) (**Figure 3F**), P53 (+ 60%) and Ki-67 (+ 80%). The following diagnosis was considered: (1) malignant tumor of the ascending colon (cT4N2M0, stage III, dMMR); (2) partial intestinal obstruction; (3) history of rectal malignant tumor; and (4) history of ileocecal malignant tumor. After discussion with the colorectal cancer multidisciplinary team (MDT), two cycles of pembrolizumab (200 mg, IV infusion, once every 3 weeks) were given from April 6 to April 27, 2023. After one cycle of treatment, the intestinal obstruction symptoms disappeared. A genetic test suggested the presence of MSI-H and LS.

**Table 1:** Main laboratory data at admission.

Parameters	Patient	Reference range
Blood routine test HGB,g/L Stool routine test Fecal occult blood Tumor marker CEA,ng/ml CA199,ng/ml Urine routine test Liver routine test Kidney routine test Blood clotting tests	104 + 1.04 9.45 Normal Normal Normal Normal	130-175 - 0-5 0-34 Normal Normal Normal Normal

HGB, hemoglobin.

**Figure 4:**
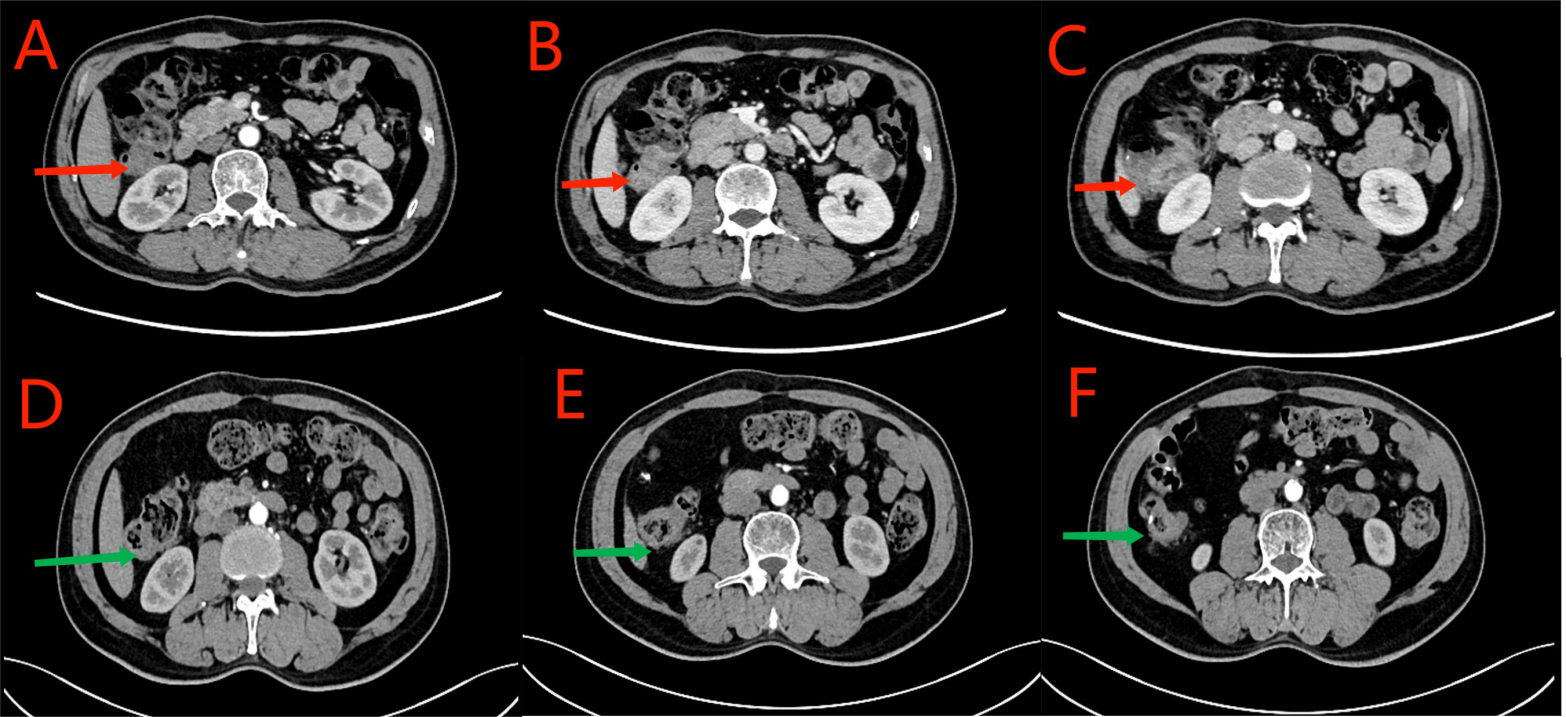
**(A–C)** CT scan conducted on May 19, 2023. Red arrows indicate segmental thickening of the anastomosis and distal intestinal wall. The tumor was smaller in size and less dense than that observed previously. Lymph nodes in the surrounding adipose space are shown; they were smaller in size than those observed previously. **(D–F)** CT scan conducted on July 21, 2023. Green arrows indicate local thickening of the anastomosis and distal intestinal wall, which was significantly reduced than that observed previously, and small lymph nodes in the surrounding adipose space.

**Figure 5:**
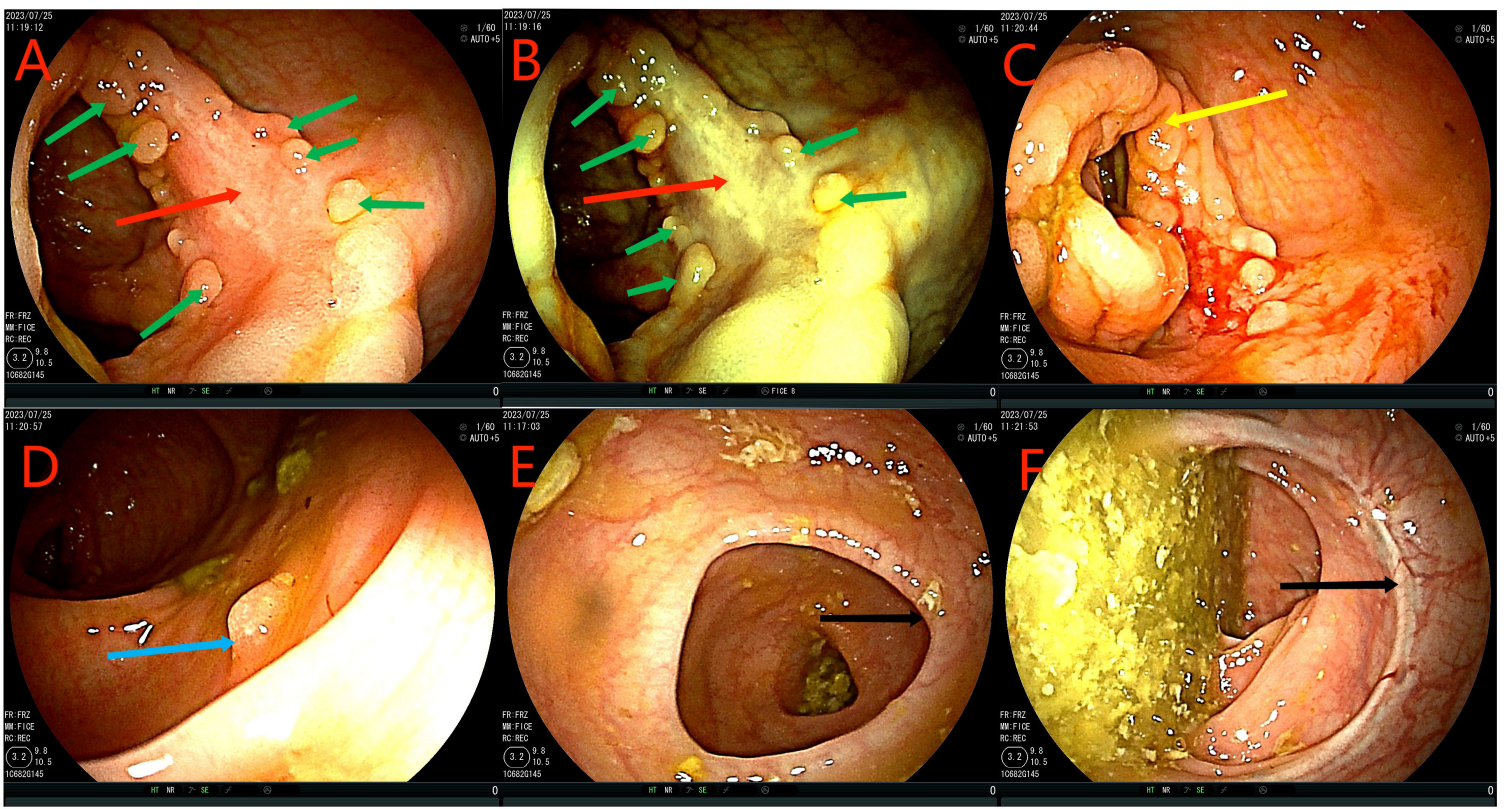
Colonoscopy examination time is shown in the upper left corner of each image. **(A, B)** The red arrow is the original tumor site, no tumor was found, and local scar like changes were observed, requiring biopsy. Green arrows show slightly rough mucosa and nodular shape. **(C)** The yellow arrow indicates the ileum at the anastomosis. **(D)** The blue arrowhead indicates intestinal adenoma. **(E, F)** Black arrows indicate ileocecal and rectal anastomoses, respectively.

**Figure 6:**
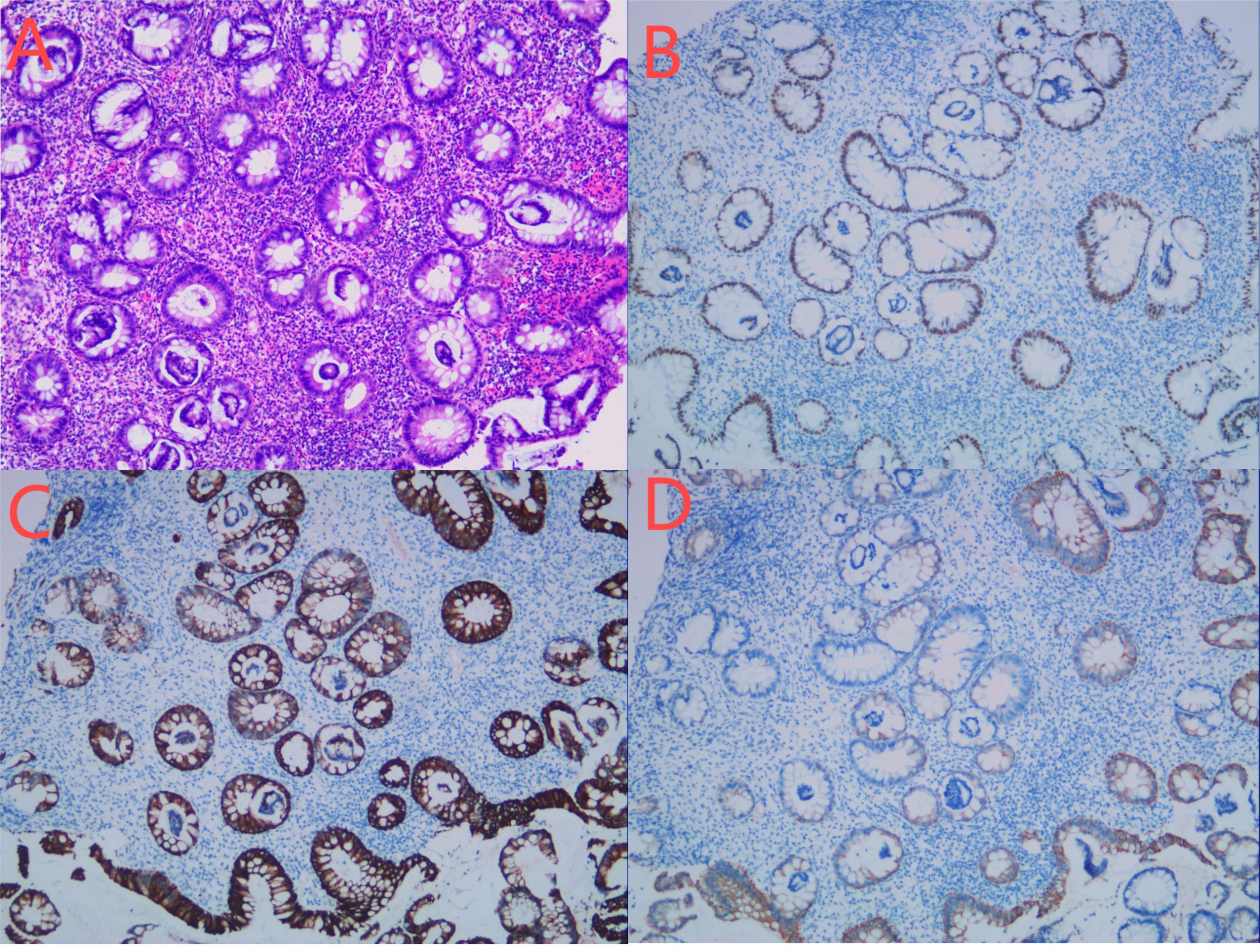
**(A–D)** Biopsy conducted on July 26, 2023. HE staining showed the morphological rules of the glands. Some glandular cells were arranged in false layers, which was consistent with the changes of tubular adenoma **(A)**. There were more inflammatory cells infiltrating in the mucous membrane lamina propria, and no clear infiltrating cancer was found. Immunohistochemical staining showed the following results: CDX2(epithelium,+) **(B)**, CK(epithelium,+) **(C)**,CK20(epithelium,+) **(D)**.

**Figure 7:**
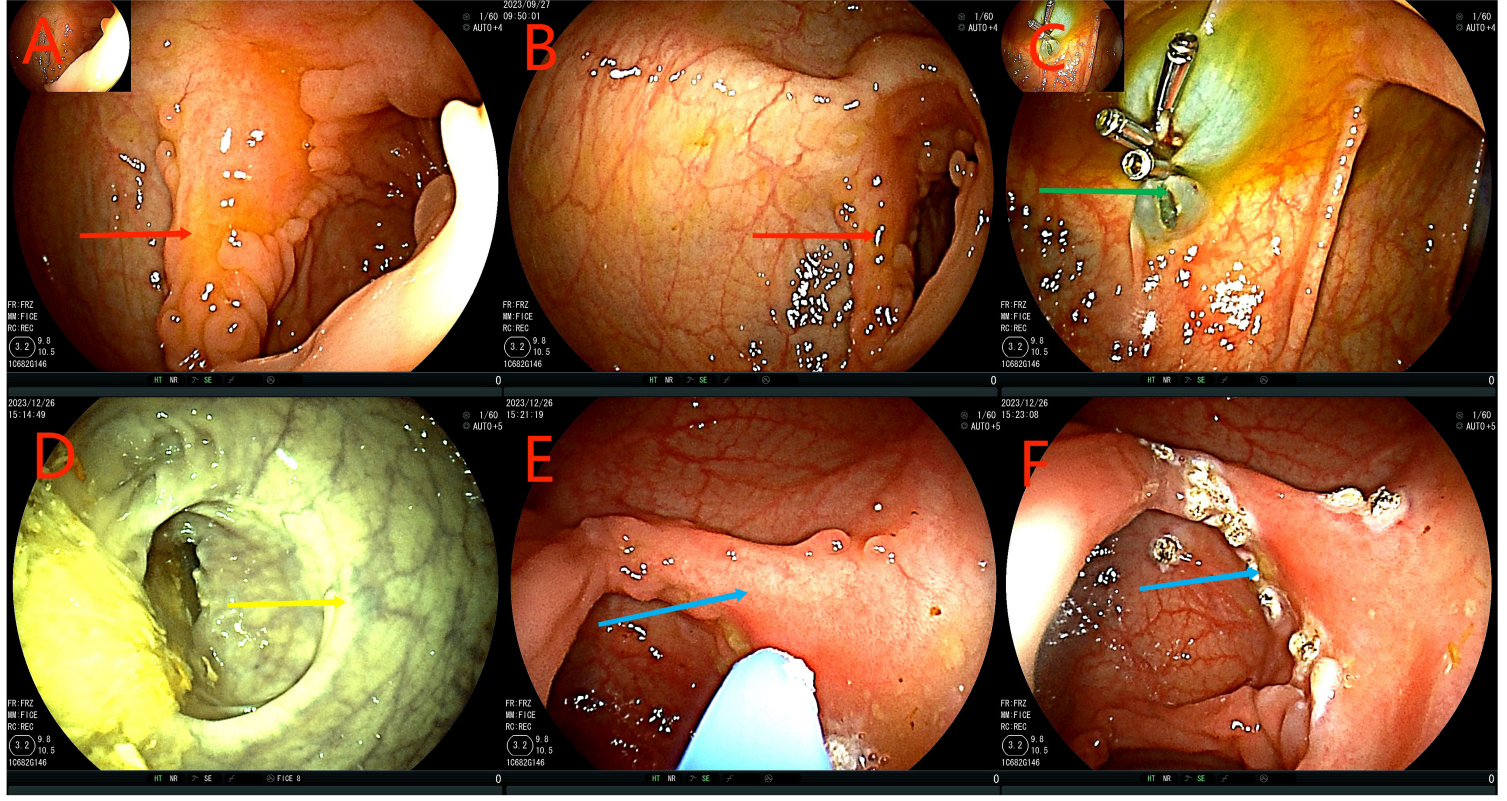
Colonoscopy examination time is shown in the upper left corner of each image. **(A–C)** Colonoscopy examination conducted on September 27, 2023. Red arrows show the ascending colon. Tumor was not detected, and local scar-like changes were observed. The green arrow shows the position after adenoma resection. **(D–F)** Colonoscopy examination conducted on December 26, 2023. The yellow arrow indicates ileocecal anastomosis. Blue arrows indicate the ascending colon. The tumor was not detected, and local scar-like changes were observed. The nodules at the primary tumor were treated by the APC.

**Figure 8:**
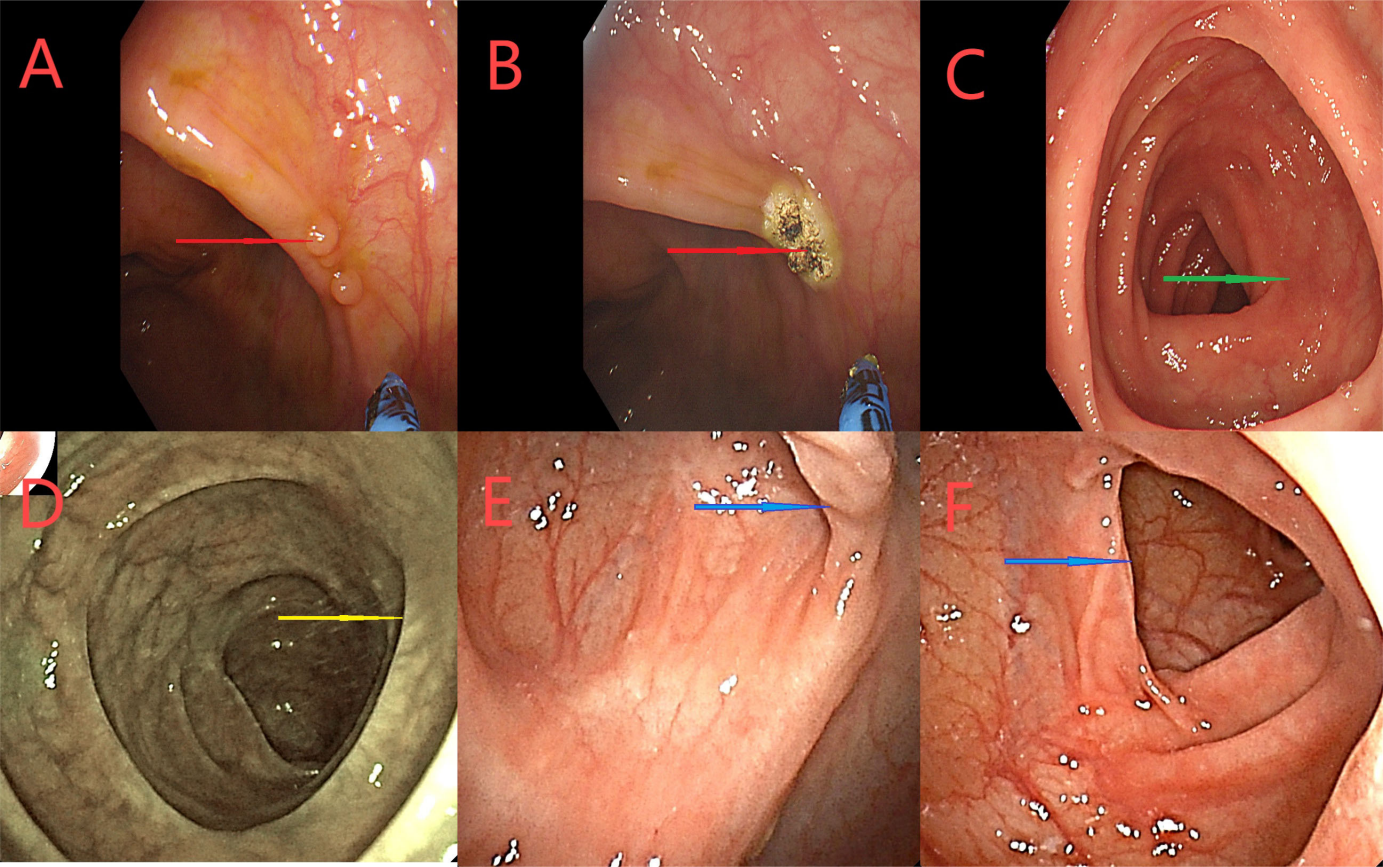
**(A–C)** A colonoscopy on March 12, 2024. Red arrows indicate the ascending nodules the original tumor site, treated the APC. Green arrows indicate the proximal small intestine. **(D–F)** Colonoscopy on June 6, 2024 Yellow arrows indicate the small intestine proximal to the ileocolic anastomosis. The blue arrows represent the original tumor site, did not see abnormalities.

**Figure 9:**
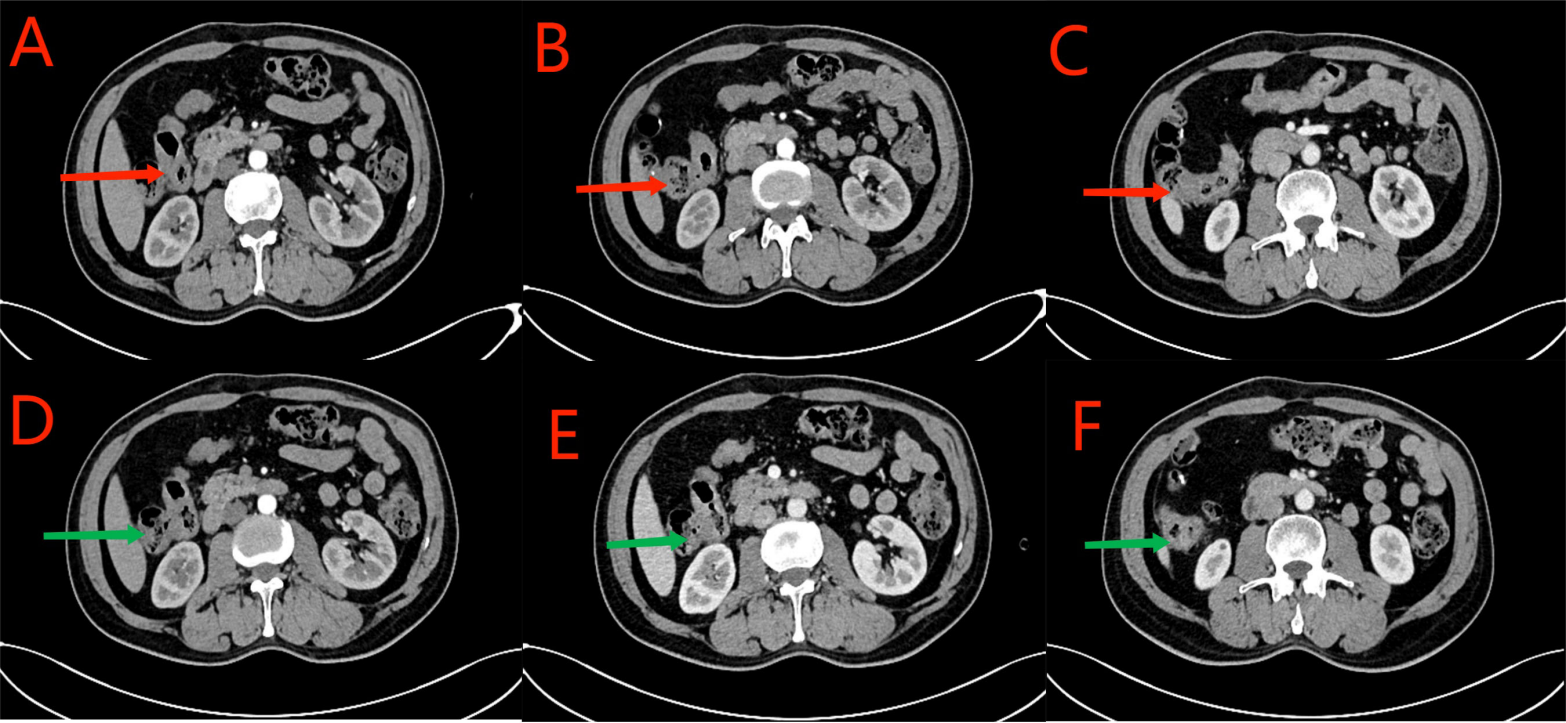
**(A–C)** CT scan conducted on September 27, 2023. Red arrows indicate local thickening of the anastomosis and distal intestinal wall; lymph nodes in the surrounding adipose space are shown. Based on the clinical data, no significant change was noted when compared with the CT scan findings of July 21, 2023. **(D–F)** CT scan conducted on December 26, 2023. Green arrows indicate local thickening of the anastomosis and distal intestinal wall; lymph nodes in the surrounding adipose space are shown. No significant change was noted when compared with the results of the CT scan conducted on September 27, 2023.

**Figure 10:**
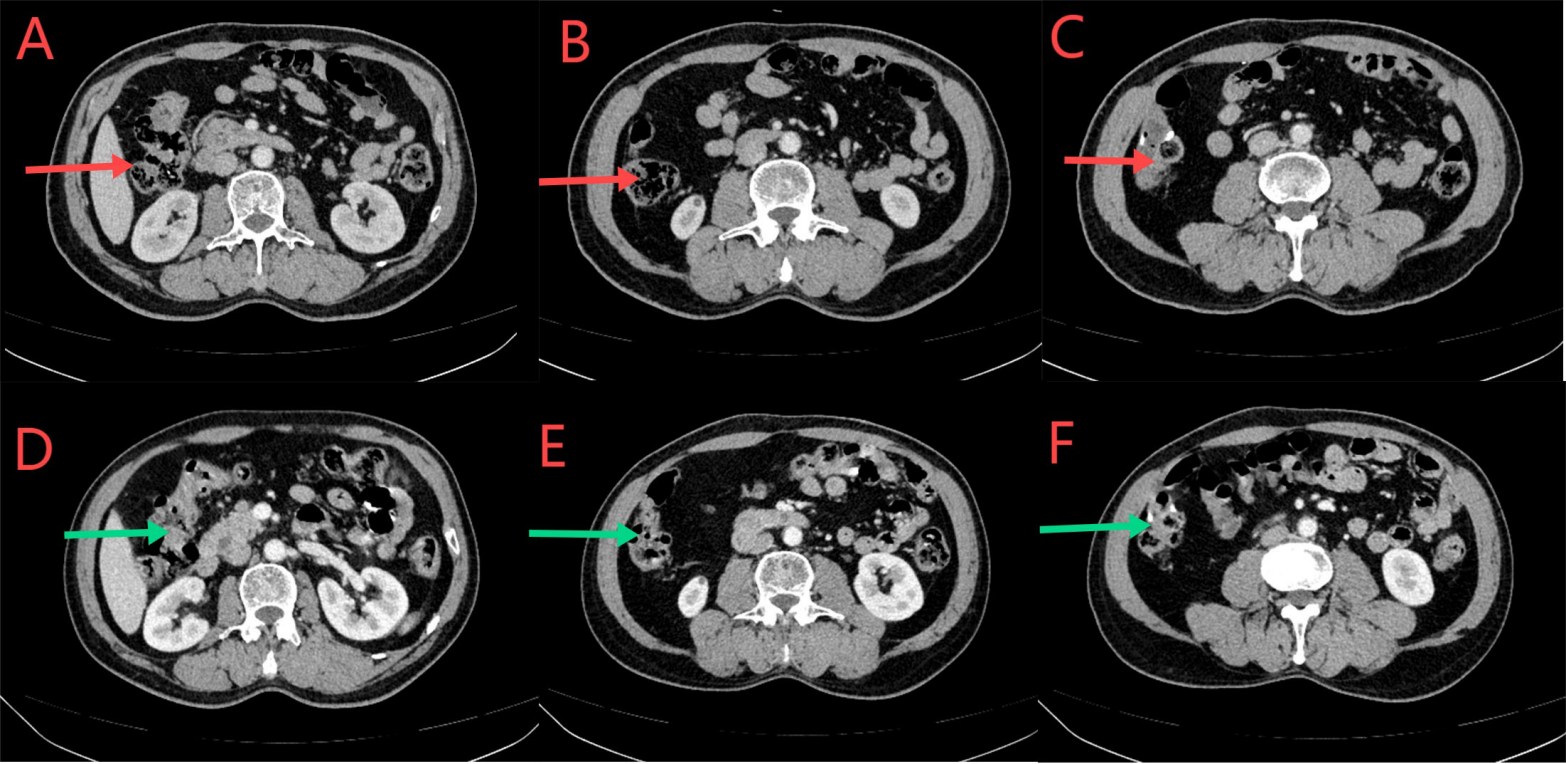
**(A–C)** CT scan conducted on March 12, 2024. The red arrows indicate normal local growth of the anastomotic and distal intestinal wall, with small lymph nodes visible in the surrounding fat space. According to the clinical data, there was no significant change compared to the CT scan on December 26, 2023. **(D–F)** CT scan conducted on June 6, 2024. Green arrows indicate local thickening of the anastomotic and distal intestinal walls; Small lymph nodes in the surrounding fat space are visible. There was no significant change compared with the CT scan on March 12, 2024.

**Figure 11:**
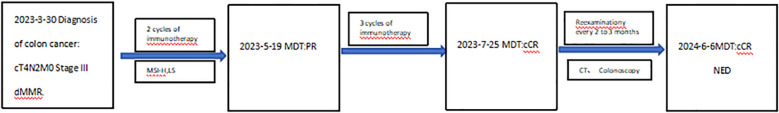
Timeline of treatment.

A reexamination CT scan conducted on May 19, 2023 showed significant tumor regression (**Figures 4A–C**). Following discussion with the MDT team, pembrolizumab (200 mg, ivgtt, q3w) was continued for additional 3 cycles. The patient was re-examined with a CT scan on July 21, 2023 (**Figures 4D–F**) and a colonoscopy examination on July 25, 2023 (**Figure 5**). Biopsy results (**Figure 6A**) indicated tubular adenoma of the ascending colon with interstitial lymphocyte aggregation. Immunohistochemical staining showed the following results: CDX2(epithelium,+) (**Figure 6B**), CK(epithelium,+) (**Figure 6C**), CK20(epithelium,+) (**Figure 6D**). Following discussion with the MDT team, complete clinical remission (ycT0N0M0, stage 0) of the tumor was confirmed. As recommended by the After discussion by the MDT team and in accordance with the patient’s wish, the W&W approach was adopted. Colonoscopy was performed on September 27, 2023, December 26, 2023, March 12, 2024, and June 6, 2024, respectively (**Figures 7**, **8A–F**), during which intestinal adenoma resection and argon plasma coagulation (APC) were performed, and no local tumor regeneration or metastasis was found in CT review (**Figures 9**, **10A–F**). We summarized the treatment of this patient, and the flow chart is shown in **Figure 11**. Up to now, the patient had a PFS of nearly 1 year.

## 3 Discussion

MSI-H/dMMR is present in the majority of solid tumors, and it is most commonly detected in endometrial, colorectal, and gastric cancers. This subtype mutation is found in 10–15% of CRC patients (4), with 20%, 11%, and 3.5% incidence in stage II, stage III, and stage IV CRC, respectively (5). In the present case, the patient previously had a stage II rectal cancer and currently had a stage III colon cancer; these findings were consistent with previous reports. Because of the defect in DNA repair, these patients are prone to the accumulation of mutations and the expression of new antigens, which may lead to the recruitment of a large number of immune cells and enhance the efficacy of immunotherapy. NICHE 1 and NICHE 2 studies showed that nivolumab combined with ipilimumab can achieve 100% postoperative pathological response, with up to 95% major pathological response (residual viable tumor: ≤10%) and 60–67% pCR (6, 7). In the PICC trial, however, the pCR rate of patients receiving toripalimab combined with the COX-1 inhibitor celecoxib was 88% (8). The MDACC study also further confirmed the efficacy of neoadjuvant immunotherapy (9). NEOCAP research proves that these patients accept card Rayleigh bead a joint resistance path for treatment of PCR reached 73% (10). At present, the new adjuvant immunization regimen in colorectal includes two types of immunotherapy, immunocombined anti-inflammatory therapy, immunocombined targeted therapy and a single immunotherapy. Combined with the current study, the combination regimen is superior to the single immunotherapy, and the PCR rate is more advantageous, but the direct comparison is lacking. In the present case, we administered pembrolizumab immunotherapy alone based on the drug efficacy, drug indication in China, and patients’ wishes. The patient achieved cCR after 5 cycles of pembrolizumab treatment, with a good response to treatment. Current studies, however, suggest that adverse effects of immunotherapy can occur in any organ system, with a variety of manifestations (11). A meta-analysis grouped the immune-related side effects of immunotherapy into three categories: general (fatigue, diarrhea, and rash), organ-specific (colitis, hepatitis, pneumonia, myocarditis, etc.), and musculoskeletal pain (arthritis and arthralgia), etc.) (12) and the most severe side effects were immune myocarditis and immune pneumonia (13). Thus far, our patient has not experienced immune-related side effects.

LS is the most common hereditary CRC syndrome with an estimated prevalence of 3% (14), and *MLH1* and *MSH2* mutations account for 60–80% of all LS-related cancers (15). Our patient had a mutation in exon 2 of the *MSH2* gene, as detected by next-generation sequencing. According to literature reports, the incidence of MSI-H/dMMR in LS and non-LS malignant tumors was 85% and 37% respectively (16), and this patient had LS with MSI-H.Partial colectomy and subtotal colectomy are frequently performed for patients with LS and colon cancer. Moreover, the incidence rate of metachronous CRC was higher after partial resection and ranged from 8% to 25% (17, 18); this incidence rate was 3.4-fold higher than that in patients undergoing subtotal colectomy, but without survival benefits (18). The analysis of the long-term results of the registry data showed that this risk increased over time, and the incidence of metachronous CRC was 16%, 41%, and 62% after 10, 20, and 30 years, respectively (19). Our patient developed three intestinal malignant tumors within a span of <10 years. Compared to subtotal colectomy, segmental colectomy has a higher incidence of anastomotic leakage (47.4% vs. 13.6%) and higher morbidity (47.4% vs. 16.6%) (20). The US Multi-Society Task Force on Colorectal Cancer has adopted colectomy combined with ileorectal anastomosis as the main surgical approach for CRC patients with LS (21). However, after subtotal colectomy, patients show a significant increase in the frequency of defecation, which negatively affects defecation-related aspects and social behavior (22); moreover, defecation cannot be easily performed after the resection of the right hemicolon (23). This is also one of the reasons why our patient refused surgical treatment.

Our patient had heterogeneous CRC, and the imaging findings indicated locally advanced colon cancer. Compared to traditional neoadjuvant chemotherapy, preoperative neoadjuvant immunization shows several advantages such as a high response rate, a high complete remission rate, and fewer side effects. Additionally, when our patient reached cCR, we used the cCR standard for reference. In this patient, endoscopy after immunotherapy revealed that the mucosa of the original lesion was slightly rough or smooth, and no tumor cells were detected in the biopsy. The CT scan showed no intestinal wall tumor and no enlarged lymph nodes. Normal levels of tumor markers were observed. After the patient achieved cCR, we recommended subtotal colon resection. However, considering that the patient had previously undergone radical resection of rectal cancer (low anastomotic position), if subtotal resection was adopted, a smaller extent of the rectum would be retained, with a higher probability of postoperative anastomotic fistula, pre-rectal resection syndrome, sexual and bladder dysfunction, and chronic diarrhea. After comprehensive consideration and the patient’s refusal to undergo surgical treatment, immunotherapy and the W&W approach were continued. According to KEYNOTE-177 (24) and other studies, the duration of immunotherapy for advanced CRC should be 2 years; however, there is no definite consensus regarding the duration of cCR achieved by neoadjuvant immunotherapy. According to the current studies, the neoadjuvant immunotherapy duration is 1–6 months (6–9). For our patient, we adopted the W&W approach after 5 cycles of neoadjuvant immunotherapy. CT and colonoscopy findings were also reviewed regularly. Thus far, the patient has not shown tumor recurrence and metastasis.

Regarding the W&W approach for rectal cancer, the rate of local tumor recurrence in the W&W group was 34%, and 88% of patients without distant metastasis received salvage surgery (25). In the W&W group, the 2-year local recurrence rate was 15.7–21.4% (26, 27), and the distant metastasis rate was 8% (27); however, 90–95.4% of patients with tumor regrowth received salvage therapy (26, 27). The R0 resection rate is 98% (27), and the 3-year overall survival rate after reoperation is 80% (27). Although the probability of local recurrence is high during the W&W period, a high R0 resection rate can be obtained by salvage surgery after local recurrence; this does not affect the overall prognosis of patients. Previous studies have shown that if a patient continues to achieve clinical complete remission for 1 year, the probability of maintaining no local tumor regrowth was 88.1% at 2 years, 97.3% at 3 years, and 98.6% at 5 years (28). For patients with clinical complete remission without distant metastasis for 1 year, the probability of distant metastasis-free survival was 93.8%, 97.8%, and 96.6% at 2, 3, and 5 years, respectively (28). Patients should preferably undergo a digital rectal examination, proctoscopy, CEA levels,MRI and CT every 8–12 weeks during the first 3 years. After these 3 years, follow-up may perhaps be loosened to every 6 months and to include primary tumor response assessment (29). We refer to colorectal cancer watchful waiting strategy, every 3 months the CT, colonoscopy and tumor markers. Thus far, our patient had 11 months of sustained clinical remission, and the probability of tumor regrowth or metastasis in the later period is low; therefore, our patient is likely to achieve complete cure. The cCR strategy is currently a safe and feasible approach; however, there is a lack of relevant research on the use of the cCR strategy for colon cancer. Based on the findings of the present case, we believe that the cCR strategy is also suitable for colon cancer, particularly for patients with LS.

## 4 Conclusion

Immunotherapy shows a good effect on CRC patients with MSI-H or dMMR gene deficiency, and most preoperative neoadjuvant immunotherapies can achieve cCR or pCR. Currently, the preservation of organ function is becoming increasingly important. In the present case, the patient’s tumor reached cCR after five cycles of neoadjuvant immunotherapy, thereby enabling the patient’s exclusion from surgery. To the best of our knowledge, the present case report is the first to report that preoperative treatment, except for rectal cancer, can achieve cCR and allow exclusion from surgery; this also provides a new direction for further clinical research and treatment. Moreover, patients with LS who require subtotal colectomy may achieve cure with neoadjuvant immunotherapy. More clinical data are required to validate that the W&W approach together with neoadjuvant immunotherapy is safe and feasible for patients with colon cancer.

## Data Availability

The original contributions presented in the study are included in the article/supplementary material. Further inquiries can be directed to the corresponding author.
